# Low genetic diversity of the Human T-cell Lymphotropic Virus (HTLV-1) in an endemic area of the Brazilian Amazon basin

**DOI:** 10.1371/journal.pone.0194184

**Published:** 2018-03-20

**Authors:** Akim Felipe Santos Nobre, Danilo de Souza Almeida, Louise Canto Ferreira, Deimy Lima Ferreira, Edivaldo Costa Sousa Júnior, Maria de Nazaré do Socorro de Almeida Viana, Ingrid Christiane Silva, Bruna Teles Pinheiro, Stephen Francis Ferrari, Alexandre da Costa Linhares, Edna Aoba Ishikawa, Rita Catarina Medeiros Sousa, Maísa Silva de Sousa

**Affiliations:** 1 Núcleo de Medicina Tropical, Universidade Federal do Pará, Belém, Pará, Brasil; 2 Instituto Evandro Chagas, Ananindeua, Pará, Brasil; 3 Department of Ecology, Universidade Federal de Sergipe, Sergipe, Brazil; George Mason University, UNITED STATES

## Abstract

The Human T-cell Lymphotropic Virus (HTLV-1) is a *Deltaretrovírus* that was first isolated in the 1970s, and associated with Adult T-cell Leucemia-Lymphoma (ATLL), and subsequently to Tropical Spastic Paraparesis-Myelopathy (TSP/HAM). The genetic diversity of the virus varies among geographic regions, although its mutation rate is very low (approximately 1% per thousand years) in comparison with other viruses. The present study determined the genetic diversity of HTLV-1 in the metropolitan region of Belém, in northern Brazil. Blood samples were obtained from patients at the UFPA Tropical Medicine Nucleus between January 2010 and December 2013. The DNA was extracted and the PX region of the HTLV was amplified using nested PCR. The positive samples were then digested using the Taq1 enzyme for the identification and differentiation of the HTLV-1 and HTLV-2. The 5’LTR region of the positive HTLV-1 samples were amplified by nested PCR, and then sequenced genetically. The phylogenetic analysis of the samples was based on the maximum likelihood method and the evolutionary profile was analyzed by the Bayesian approach. Overall, 78 samples tested positive for HTLV-1, and 44 were analyzed here. The aA (cosmopolitan-transcontinental) subtype was recorded in all the samples. The following evolutionary rates were recorded for the different subtypes–a: 2.10−^3^, b: 2.69. 10−^2^, c: 6.23. 10−^2^, d: 3.08. 10−^2^, e: 6. 10−^2^, f: 1.78. 10−^3^, g: 2.2. 10−^2^ mutations per site per year. The positive HTLV-1 samples tested in the present study were characterized by their low genetic diversity and high degree of stability.

## Introduction

The Human T-cell Lymphotropic Virus (HTLV-1) is a *Deltaretrovirus* that was first isolated from a blood sample taken in the 1970s from an African-American patient with a cutaneous T-cell lymphoma [1]. The lymphoma was subsequently classified as Adult T-cell Leucemia-Lymphoma (ATLL), a severe disease that affects the lymphocytes. The virus was subsequently related to Tropical Spastic Paraparesis-Myelopathy (TSP/HAM), a chronic and progressive disease that affects primarily the thoracic spinal medulla [2].

It has been estimated that, worldwide, 15 to 20 million are infected with HTLV-1 [3]. The virus is endemic in southern Japan, the Caribbean, Central Africa, Central and South America, Melanesia, and the aboriginal population of Australia, with a seroprevalence of 1–30%. Sporadic HTLV-1 also occurs in risk groups in non-endemic areas, including major cities in the United States and western Europe [4].

In Brazil, HTLV-1 was first detected in Japanese immigrants living in Campo Grande, in the state of Mato Grosso do Sul, in 1986 [5], and most of the epidemiological data on this infection in the country refer to its prevalence in specific populations. In blood donors, a geographic gradient has been observed, with a lower prevalence in the south of the country, rising towards the north [6]. The only population-level study in Brazil focused on the city of Salvador, capital of the state of Bahia [7], where a prevalence of 1.8% was recorded in the general population, with much lower rates in males (1.2%) compared with females (2.0%), and much higher rates in individuals of over 50 years of age (6.3% in females and 9.0% in males).

Diseases associated with HTLV-1 infection have been reported from a number of Brazilian regions [8]. In Bahia, cases of TSP/HAM, scabies, and strongyloidiasis were recorded in individuals infected with HTLV-1, and patients infected with this virus also presented a higher prevalence of tuberculosis. As in other countries, then, this infection tends to be more prevalent in socially underprivileged groups, in particular poorly-educated women [9].

The HTLV-1 virus replicates slowly, and studies are under way for the development of a vaccine [10]. Few data are available from the Amazon region, however, reinforcing the need for more detailed and representative studies of the infected population to support research into possible vaccines and antiviral treatments for HTLV-1.

As this virus is associated with highly deleterious diseases and is endemic to the Amazon region, one major priority is to investigate its epidemiology and genetic variability in the metropolitan region of the city of Belém, the capital of Pará state, and the largest urban center in northern Brazil [11,12, 13, 14].

## Materials and methods

### Type of study

The present study was transversal and retrospective.

### Study population

The study was based on the analysis of samples taken from seropositive patients for HTLV-1/2, as confirmed molecular procedures (PCR) for HTLV-1. These patients were treated at the Tropical Medicine Nucleus at the Federal University of Pará (UFPA), in Belém, Brazil, between January 2010 and December 2013.

#### Inclusion criteria

The study included patients of both sexes and all ages treated at the UFPA Tropical Medicine Nucleus during the study period. The study prioritized patients that agreed to participate in the research and had signed a term of informed consent.

#### Exclusion criteria

All samples considered inadequate for analysis were excluded from the study.

#### Ethical questions

The present study was approved by the Committee for Ethics in Research of the UFPA Tropical Medicine Nucleus, under protocol CAAE 31014114.2.0000.5172. All the norms and regulations of the UFPA were followed rigorously.

### Extraction of the proviral DNA

Aliquots (5 mL) of blood were collected in tubes containing EDTA, with the leukocytes being separated from the plasma (mononuclear cells of the peripheral blood) and stored at -20°C until analysis. The proviral DNA was extracted from the layer of leukocyte cells following centrifugation of the total blood, using a Wizard®Genomic DNA Purification kit (Promega, Madison WI, USA) according to the maker’s instructions.

### Amplification of the human *β* globin (internal reaction control)

The human β globin was amplified to evaluate the integrity of the genetic material extracted. The amplification was based on the oligonucleotides G73 (5’ GAAGAGCCAAGGACAGGTAC-3’) and G74 (5’-CAACTTCATCCACGTTCACC-3’) [15].

### Amplification and genotyping of the HTLV

Once the proviral DNA was amplified, a nested PCR was run, followed by enzymatic digestion for the confirmation of the HTLV infection and the differentiation of the virus types 1 and 2. For this, the pX region of the virus was amplified. This initial reaction was run in a solution containing 5.0 de Go Taq Green Master Mix (Promega, Madison, WI, USA), 2.0 μL of water, 1 μL (10 pmol) of each primer–HTLV_External F 5'-TTCCCAGGGTTTGGACGAAG-3' (7219–7238, forward) and HTLV_External R 5'-GGGTAAG GACCTTGAGGGTC-3' (7483–7464, reverse)–and 1.0 μL of the DNA, for a final volume of 10 μL.

In the second phase of the nested PCR, the same quantity of Go Taq Green Master Mix (Promega, Madison, WI, USA) was used, together with 1μL (10 pmol) of the primers HTLV_internal F 5'CGGATACCCAGTCTACGTGTT3' (7248–7268, forward) and HTLV_internal R 5'GAGCCGATAACGCGTCCATCG3' (7406–7386, reverso), 2.5 μL of water and 0.5 μL of the product of the first PCR, producing a fragment of 159 bps [16].

Positive (a sample known to be infected) and negative controls were used for each PCR reaction. The products were electrophoresed (60 minutes at 100 V) in 2% agarose gel, stained with ethidium bromide in 1x TAE buffer (TAE 50x stock—1.6 M Tris-Base, 0.8 M sodium acetate, and 40 mM EDTA-Na2 per liter of deionized water), and visualized using an ultraviolet transilluminator.

### Enzymatic digestion for the identification of the type of HTLV

To identify the HTLV type (1 or 2), the positive cases identified by PCR were processed by enzymatic digestion for the identification of RFLPs (Random Fragment Length Polymorphism). The RFLP reaction of the product of the pX gene (159 bps) was conducted in a mixture of 6.0 μL of the amplified product, 7 μL of H_2_O, 1.5 μL of buffer (Promega, Madison WI, USA), and 0.5 μL of the TaqI 10 U/μL restriction enzyme (Promega), which was incubated for 2 hours at 65°C.

The digestion of the restriction site (T/CGA) generates three fragments of 85 bps, 53 bps, and 21 bps in HTLV-2, and two (138 bps and 21 bps) in HTLV-1. The products of the digestion were electrophoresed in 3% agarose gel with 1x TAE buffer (as above) for visualization using an ultraviolet transilluminator.

### Amplification of the 5'LTR region of the HTLV-1

The 5'LTR region of the HTLV-1 was amplified in a total volume of 25 μL, containing 500 ng of the extracted DNA, 125 μM of each dNTP, 10 pmol/μL of each primer, 3.0 μM of MgCl_2_, 50 mM of KCI, 10 mM Tris-HCI pH 8.3, and 0.1 U of Taq DNA polymerase. The primers used to amplify the HTLV-1 were (LTR-I.01) 5'-TGACAATGACCATGAGCCCCAA-3' and (LTR-I.02) 5'¬CGCGGAATAGGGCTAGCGCT-3', corresponding to nucleotides 1–22 and 823–842 of the ATK HTLV-1 strain [17].

In the second step of the nested PCR, 2.0 μL of the product of the first step (see above) was analyzed using primers internal to the region amplified previously. The primers were (LTR-I.03) 5'-GGCTTAGAGCCTCCCAGTGA-3'/ (LTR-I.04) and 5'-GCCTAGGGAATAAAGGGGCG-3', corresponding to nucleotides 30–49 and 781–800 of the HTLV-1ATK strain [17]. The amplification protocol, following initial denaturation for 5 minutes at 94°C, involved 35 cycles of 40 seconds at 94°C, 30 seconds at 60°C, and 90 seconds at 72°C, followed by a final extension of 10 minutes at 72°C, which produced a fragment of 744 bps. The products of this amplification were electrophoresed (60 minutes at 100 V) in 2% agarose gel stained with ethidium bromide in 1x TAE buffer (as above) and visualized using an ultraviolet transilluminator. The samples that presented the 744 bp amplicon were purified from the product of the nested PCR using the QIAquick PCR Purification kit (QIAGEN, Inc, USA), following the maker’s instructions.

### Pre-sequencing PCR

A Big Dye® terminator Cycle Sequencing kit (Applied Biosystems, Inc, Foster City, CA, USA) was used for the sequencing reaction. The reaction mixes had a final volume of 10 μL, composed of 2 μL de Big Dye, 1 μL of buffer, 2 μL of the DNA (50 ng), and 5 pmol/μL de each primer (as in the second step of the PCR of the ‘5 LTR), and 3 μL of deionized water (when necessary) in individual reactions. This mixture was processed in an automatic thermocycler (Master Cycler, Eppendorf, Birkmann Instruments), with an initial cycle of 2 minutes at 94°C, followed by 25 cycles of 45 seconds at 94°C, 30 seconds at 50°C, and 4 minutes at 60°C.

### Electrophoresis of the sequenced DNA

Following the purification of the PCR product (5 'LTR region), the DNA was sequenced by the Sanger *et al*., 1977 [18] method of biochemical synthesis of the DNA, using an ABI PRISM Big Dye Terminator Cycle Sequencing kit (Applied Biosystems, Inc, Foster City, CA, USA).

### Edition and alignment of the sequences

The nucleotide sequences were analyzed and edited in GENEIOUS 4.8.5, and aligned with sequences of other viruses available in GenBank (http://www.ncbi.nlm.nih.gov), using MAFFT, v. 7.

### Analysis of the sequences and construction of the phylogenetic tree

The distance matrix and phylogenetic tree were constructed in IqTREE, using the maximum likelihood method. The bootstrap analysis was based on 1000 replicates to ensure the most reliable estimate of the relationships among the different groups, with the procedure being repeated 10 times. The tree was edited in FigTree 1.4.2 [19].

### Estimates of the rate of genetic diversification in HTLV-1

The diversification of the sequences was evaluated in comparison with the sequences available in GenBank, with the rate of diversification being estimated in BEAST v. 1.8, based on 200 samples containing all the HTLV-1 subtypes, and a total of 300 million comparative trees. This analysis was based on Bayesian inference [20].

## Results

**Fig 1:**
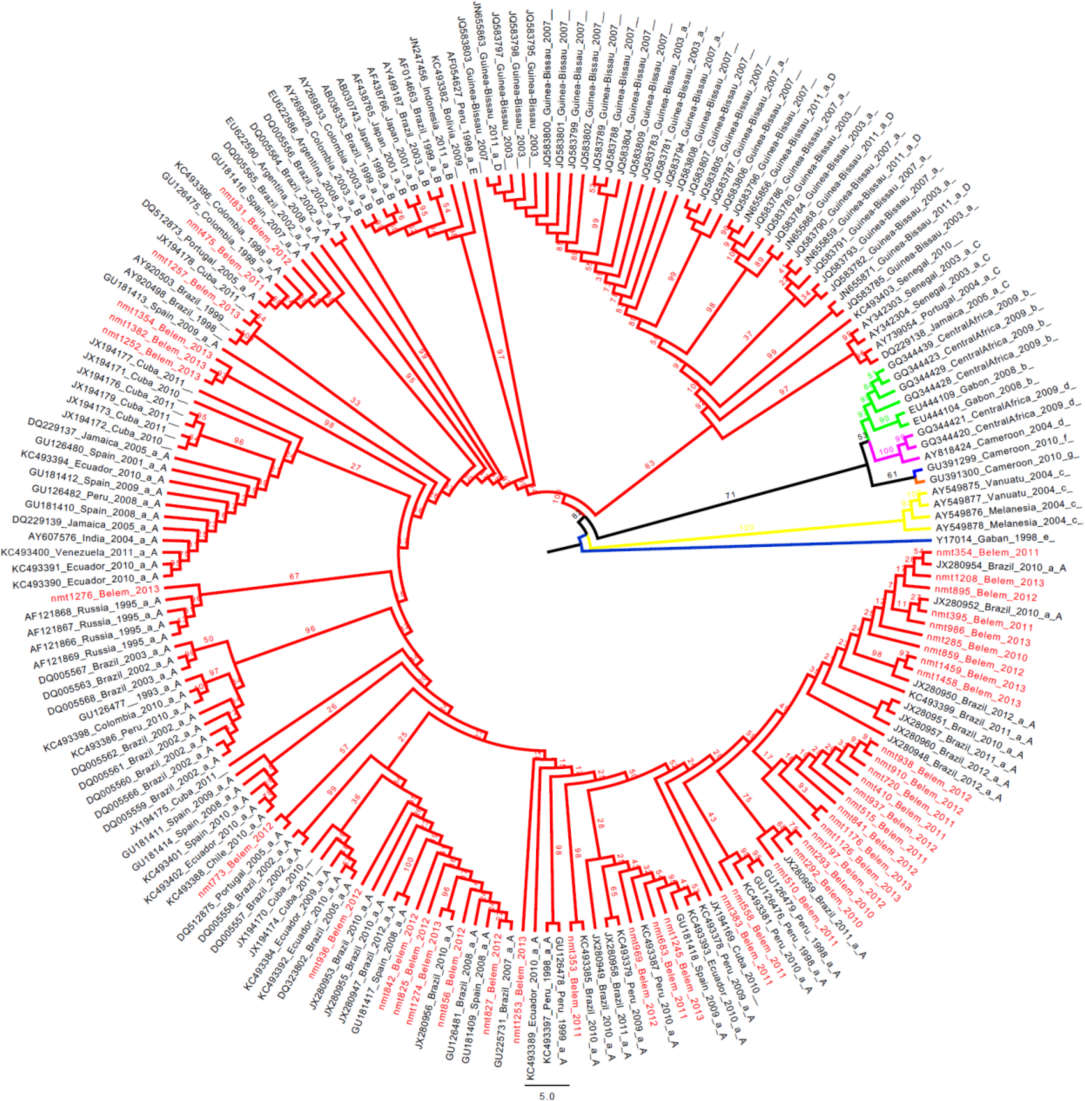
Phylogenetic analysis of the 744 bps nucleotide sequence corresponding to the 5’LTR region of the HTLV-1, based on the maximum likelihood method with 10,000 bootstrap replicates. The samples analyzed in the present study are shown in red, and all other were obtained from GenBank (https://www.ncbi.nlm.nih.gov/genbank).

Overall 78 of the samples obtained from the UFPA Tropical Medicine Nucleus between January 2010 and December 2013 tested positive for HTLV-1. More than two-thirds (55 or 70.5%) of these samples were obtained from female patients, with only 23 (29.5%) from male patients. The mean age of these patients was 44 years old. It was possible to sequence the 5’LTR region of 44 of these 78 samples. All the samples presented the HTLV-1 aA (transcontinental cosmopolitan) genotype (see Fig 1).

The genetic diversity of the 44 sequences obtained in the present study (GenBank access numbers MF384328—MF384371) was compared with that of 200 HTLV-1 samples representing the seven different HTLV-1 subtypes. A total of 300 million comparative trees were generated, revealing the following rates of evolution for the different subtypes–a: 2.10−^3^, b: 2.69. 10−^2^, c: 6.23. 10−^2^, d: 3.08. 10−^2^, e: 6. 10−^2^, f: 1.78. 10−^3^, g: 2.2. 10−^2^ mutations per site per year (S1 Fig and S1 Table).

Only 50 of the 78 patients that tested positive for HTLV-1 were monitored subsequently in the outpatients ward of the UFPA Tropical Medicine Nucleus. Of these patients, 16 (32%) presented symptoms of TSP/HAM, while the other 34 (68%) were asymptomatic.

## Discussion

The HTLV-1 virus has a worldwide distribution, with considerable variation among regions in the prevalence of infection. A number of studies have highlighted the importance of historic migrations and miscegenation for the dispersal of this virus, which now infects approximately 20 million people worldwide. The present study confirmed that the aA HTLV-1 genotype is found in the metropolitan region of Belém, which is consistent with the evidence from other regions of Brazil and Latin America.

The aA subtype is one of the most common HTLV-1 genotypes, especially in populations that have a long history of migration, as in northern Brazil. This may at least partly account for the high frequency of this genotype recorded in the metropolitan region of Belém, given that a similar profile of infection has been recorded in other, comparable urban environments in developing countries, in particular in Latin America [2, 4].

In the present study, 32% of the patients were symptomatic, a relatively high rate in comparison with previous studies. This may be linked directly to the sample population, given that the outpatients department of the UFPA Tropical Medicine Nucleus attends many blood donors with positive serology for HTLV and other individuals with symptoms indicative of infection by this virus, but with unknown etiology. One other possible factor is the relatively high mean age of the infected individuals (44 years), which may contribute to the manifestation of the infection after years of latency, in addition to the immunological characteristics of the patient.

All the sample that were positive for HTLV-1 were included in the subtype aA clade in the phylogenetic tree, together with samples from Colombia, Spain, Russia, Jamaica, Ecuador, Peru, Portugal, Japan, Senegal, and other countries, including Brazil. Some of the 44 samples analyzed here were tightly grouped, reflecting a high degree of conservation and similarity of the virus being disseminated through the region during the study period, which may also indicate the evolution of a new HTLV-1 a subgroup, a hypothesis that will require further, confirmatory data.

The molecular investigation of HTLV-1 has permitted the identification not only of the origin of this retrovirus, but also its dispersal among the different human populations around the world. Rego et al. 2008 [21] analyzed the prevalence of infection and molecular epidemiology of the virus in three populations (Taquarandi, Junco and Alegre) of the basin of the São Francisco River, in Bahia, finding a low genetic diversity, similar to that recorded in the present study. The molecular data indicate that the virus has been introduced into Brazil in multiple events, and may even have a pre-Columbian origin [21].

Other studies have also shown that HTLV-1 is relatively stable, and has low genetic diversity in comparison with other retroviruses [22]. Mutation rates range between 3.44 x 10^−7^ and 6.55 x 10^−7^ substitutions per site per year for the env and LTR, respectively [23]. A substitution of approximately 1% per thousand years has also been reported [24, 25]. This genetic stability is probably related to the viral replication strategy [26], although the degree of genetic polymorphism of the different virus subtypes is closely linked to their geographic origin [23, 25, 27, 28, 29]. In the case of the HTLV-1, the different subtypes diverge by 2–8%, although the genetic variation found in a given subtype is less than 0.5% [25]. In the HTLV-2, however, this variation reaches 4.3% in the env region when compared with subtypes a and b [30].

The phylogenetic tree revealed a rate of 2. 10−^3^ mutations per site per year for the subgroup a samples, which included all 44 analyzed in the present study. This is an extremely low rate in comparison with the evolution of the rotavirus, and the smallpox and influenza viruses. A curious detail here is that, while vaccines are available for these much more variable viruses, no vaccine has yet been developed for the HTLV-1, despite its low mutation rates.

The phylogenetic tree was relaxed, comparing all the HTLV-1 subtypes, and it would have been expected to indicate much higher evolutionary rates, given the inclusion of highly divergent subtypes, such as c, e, f and g. However, mutation rates were low, even in these subtypes. Lemey *et al*. 2005 [23] applied a similar comparative method to that used in the present study, and found even lower rates of evolution, although the analysis included only a single sample of each group. The fact that the present study included a total of 200 samples may, in part, account for the slightly higher rates recorded here.

Prevention and control are the two primary strategies for the prevention of infection by HTLV-1, and the associated vulnerability of the sufferers to highly debilitating diseases, which currently have no effective treatment. The investigation of new cases in endemic regions, such as the Amazon basin, as in the present study, will be the principal means of tracking the progress of this virus.

## Supporting information

S1 Fig Phylogenetic tree showing rates of evolution of HTLV-1 according to each subtype. The rates (estimated in mutations per site per year) were calculated by Bayesian inference. The 44 samples sequenced in this study are shown in the tree in red letters.
(TIF)

S1 Table Table showing the evolution rate (in mutations per site per year) for each subtype of HTLV-1, obtained by Bayesian inference. (DOCX)
