# Supplementary figures and images for: Low genetic diversity of the Human T-cell Lymphotropic Virus (HTLV-1) in an endemic area of the Brazilian Amazon basin
Source: PLoS One. 2018 Mar 20;13(3):e0194184. doi: 10.1371/journal.pone.0194184 (PMC5860735; doi:10.1371/journal.pone.0194184)

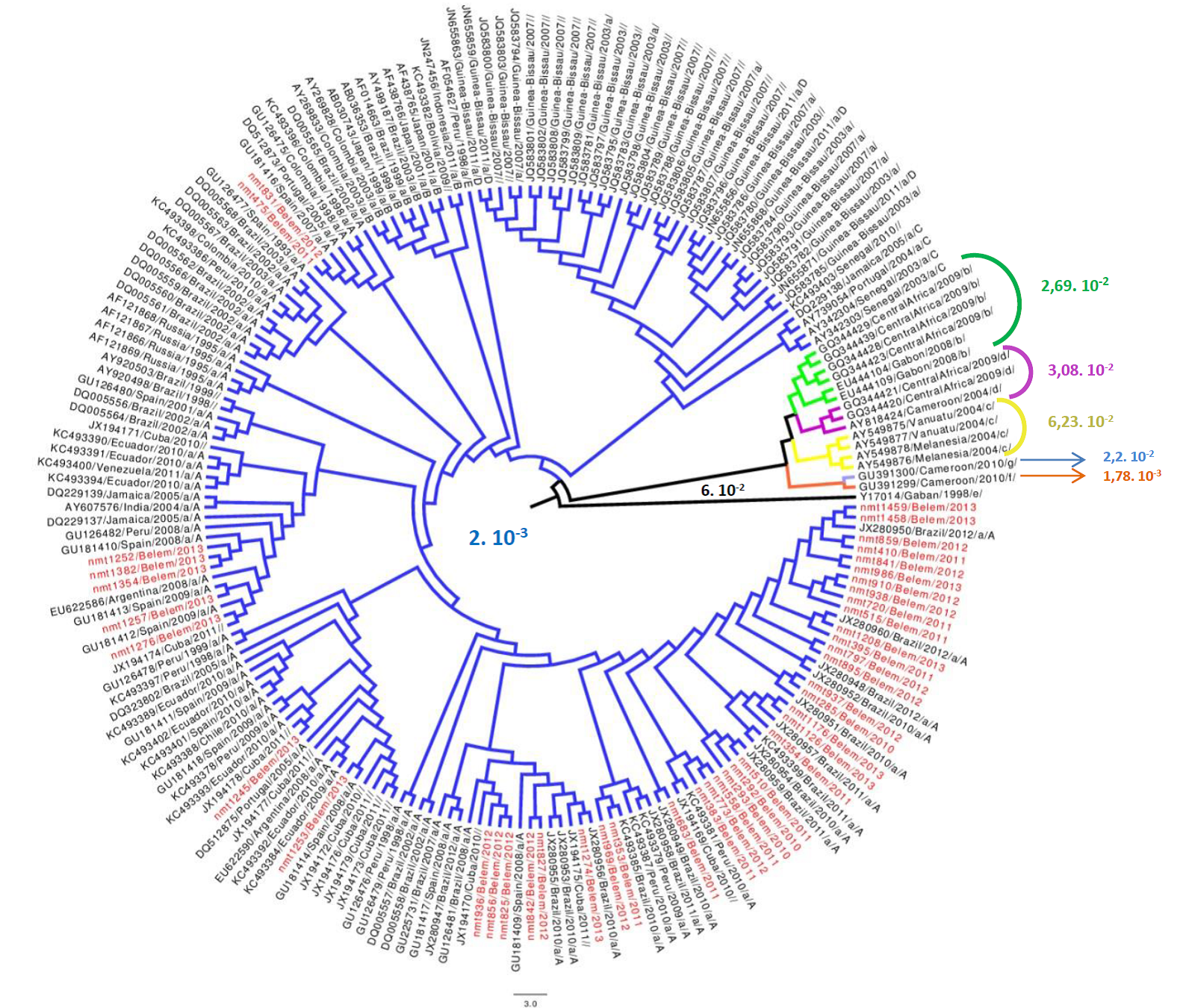

Supplement: S1 Fig — The rates (estimated in mutations per site per year) were calculated by Bayesian inference. The 44 samples sequenced in this study are shown in the tree in red letters. (TIF) [file pone.0194184.s001.tif]
