# Supplementary material for: Low genetic diversity of the Human T-cell Lymphotropic Virus (HTLV-1) in an endemic area of the Brazilian Amazon basin
Source: PLoS One. 2018 Mar 20;13(3):e0194184. doi: 10.1371/journal.pone.0194184 (PMC5860735; doi:10.1371/journal.pone.0194184)
Supplement: S1 Table — (DOCX) [file pone.0194184.s002.docx]

| **Subtyps** | **a** | **b** | **c** | **d** | **e** | **f** | **g** |
| --- | --- | --- | --- | --- | --- | --- | --- |
| **Evolution Rates** | **2 . 10 ^-3^** | **2,69 . 10 ^-2^** | **6,23 . 10 ^-2^** | **3,08 . 10 ^-2^** | **6 . 10 ^-2^** | **1,78 . 10 ^-3^** | **2,2 . 10 ^-2^** |
